# Arsenic trioxide and all-trans retinoic acid (ATRA) treatment for acute promyelocytic leukemia in all risk groups: study protocol for a randomized controlled trial

**DOI:** 10.1186/s13063-018-2812-3

**Published:** 2018-09-05

**Authors:** Xinxin Zhang, Huiyun Zhang, Limei Chen, Mengchang Wang, Jieying Xi, Xin Liu, Ming Xie, Dengzhe Li, Ekamjyot Singh Gulati, Sha Gong, Huaiyu Wang

**Affiliations:** Department of Hematology, First affiliated hospital of Xi’an Jiaotong University, Yanta West Road, Xi’an, 710061 Shaanxi China

**Keywords:** Acute promyelocytic leukemia, Arsenic trioxide, All-trans retinoic acid, Hydroxyurea, Mannitol

## Abstract

**Background:**

The treatment of acute promyelocytic leukemia (APL) has been revolutionized in the past two decades by the advent of all-trans retinoic acid (ATRA) and arsenic trioxide (ATO). It suggests that non-high-risk APL patients can be cured without chemotherapy. However, ATRA plus chemotherapy is still the standard therapy for the high-risk patients. Central nervous system (CNS) relapse remains a significant cause of treatment failure in high-risk patients. However, increasing the ATO concentration in cerebrospinal fluid (CSF) may reduce CNS relapse in high-risk patients. Mannitol can allow ATO to penetrate the blood-brain barrier (BBB) and reach therapeutically effective levels in the CSF. It is used for the treatment of CNS relapse in patients APL. We compare ATRA-ATO with ATRA-ATO plus chemotherapy in both high-risk and non-high-risk patients with APL.

**Methods:**

This study was designed as a multicenter randomized controlled trial. Patients with APL were randomly assigned into two groups: the ATRA-ATO group (experimental group) and the ATRA-ATO plus chemotherapy group (control group). The experimental group receives therapy with ATRA-ATO for induction, consolidation and maintenance therapy. In the high-risk patients, mannitol will be used with ATO in the consolidation and maintenance therapy. Hydroxyurea will be used in patients who developed leukocytosis in the induction therapy. The control group receives therapy with ATRA-ATO plus chemotherapy for induction and consolidation therapy.

**Discussion:**

In this study, a randomized clinical trial design is described. It aims to compare the efficacy of ATRA-ATO versus ATRA-ATO plus chemotherapy in all-risk patients with APL.

**Trial registration:**

Chinese Clinical Trials Registry, ID: ChiCTR-IPR-15006821. Registered on 27 July 2015.

**Electronic supplementary material:**

The online version of this article (10.1186/s13063-018-2812-3) contains supplementary material, which is available to authorized users.

## Background

Acute promyelocytic leukemia (APL) is a unique subtype of acute myeloid leukemia (AML) which accounts for 10–15% of acute myeloid leukemia. It is characterized by the *PML-RARα* fusion gene generated by the t (15; 17) (q22; q21) chromosomal translocation. Historically, APL was considered to be one of the fatal types of acute leukemia with a high risk of disseminated intravascular coagulation (DIC) and early hemorrhagic death. The application of all-trans retinoic acid (ATRA) [1, 2] and arsenic trioxide (ATO) [3–5] modifies APL from being highly fatal to highly curable. The APL0406 study showed that the advantages of ATRA-ATO over ATRA chemotherapy increase over time and that there is significantly greater and more sustained antileukemic efficacy of ATRA-ATO compared with ATRA chemotherapy in non-high-risk APL patients [6, 7]. For high-risk patients, ATRA plus chemotherapy is, however, still the standard therapy. Recently, Burnett et al. [8] compared ATRA-ATO with ATRA plus chemotherapy in both high-risk and low-risk patients with APL. They confirmed that ATRA-ATO is a feasible treatment in low-risk and high-risk patients with APL, with a high cure rate and less relapse than, and survival not different to, ATRA and idarubicin. High-risk patients in the treatment received an initial dose of the immunoconjugate gemtuzumab ozogamicin. But immunoconjugate gemtuzumab ozogamicin is not available in many countries. Wang et al. [9] showed that a mannitol infusion can cause a transient increase in blood-brain barrier (BBB) permeability to ATO, thereby increasing the ATO concentration in the CSF and curing most patients with CNS relapse. We designed a multicenter randomized controlled trial, to prove that the treatment of ATO combined with ATRA is possibly superior to ATO combined with ATRA and chemotherapy, for all levels of risk in APL patients. In this trial, mannitol will be provided to high-risk patients to prevent CNS relapse.

## Methods

### Study hypothesis

To prove that ATRA-ATO is not inferior, and may be superior to, ATO-ATRA with conventional chemotherapy, not only for non-high-risk but also for high-risk APL patients.

### Eligibility criteria

Patients will be enrolled from the following hospitals: The First Affiliated Hospital of Xi’an Jiaotong University, Baoji Central Hospital, Shanxi People’s Hospital, Xi’an Central Hospital, The Second Affiliated Hospital of Xi’an Jiaotong University, Chinese People’s Liberation Army Hospital No. 323, and The Second Affiliated Hospital of Yan’an University.

All study participants will sign a written informed consent before participation. All participants will go through a standardized interview process and receive more information about the study and the treatments. The purpose, procedures, potential risks and benefits of the study will also be explained thoroughly to the participants. The participants will be able to withdraw from the study at any time without consequence.

### Inclusion criteria

Participants meeting the following criteria will be included:

1. Age 15 to 80 years
2. Newly diagnosed APL by cytomorphology, confirmed also by molecular analysis
3. Patients who can complete the entire treatment process
4. Serum total bilirubin ≤ 3.0 mg/dL (≤ 51 μmol/L)
5. Serum creatinine ≤ 3.0 mg/dL (≤ 260 μmol/L)
6. White blood cell (WBC) count at diagnosis < 40 × 10^9^/L
7. Signed written informed consent provided

### Exclusion criteria

Participants meeting one or more of the following criteria will be excluded:

1. Allergy to the drug ingredient, the supplementary material
2. Cardiac insufficiency, renal insufficiency, significant arrhythmias, electrocardiogram (EKG) abnormalities or other important organ dysfunction
3. Other active malignancy at time of study entry
4. Pregnant or lactating women
5. Concomitant severe psychiatric disorder

### Sample size calculation

The main endpoint of the study is the disease-free survival (DFS) probability at 2 years after diagnosis. Any of the following events will be considered a failure: no achievement of hematological complete remission after induction therapy, relapse or death.

Based on the academic literature, the 2-year DFS in the experimental group is expected to be 97%, and in control group is expected to be equal to 95%. In this trial, we chose an entry time of 24 months and the total research time is 36 months. The sample size is calculated by non-inferiority testing, (one-sided *α* = 0.05, *β* = 0.20). The non-inferiority or superiority margin is − 0.1. The sample size was calculated by using PASS software (NCSS, Kaysville, UT, USA), 27 evaluable patients per group were required to draw a non-inferiority conclusion. To allow for withdrawal, we plan to recruit 72 patients, randomly allocating them into the two arms (1:1). Among each group, 12 high-risk patients and 24 non-high-risk patients will be enrolled. We will not plan an interim analysis but we will plan a final analysis when all of the 72 required patients complete 2 years of follow-up. Non-inferiority will be concluded if the lower limit of the 95% confidence interval (CI) for the rate difference of DFS is greater than 10% of the non-inferiority margin.

### Randomization

**Fig. 1:**
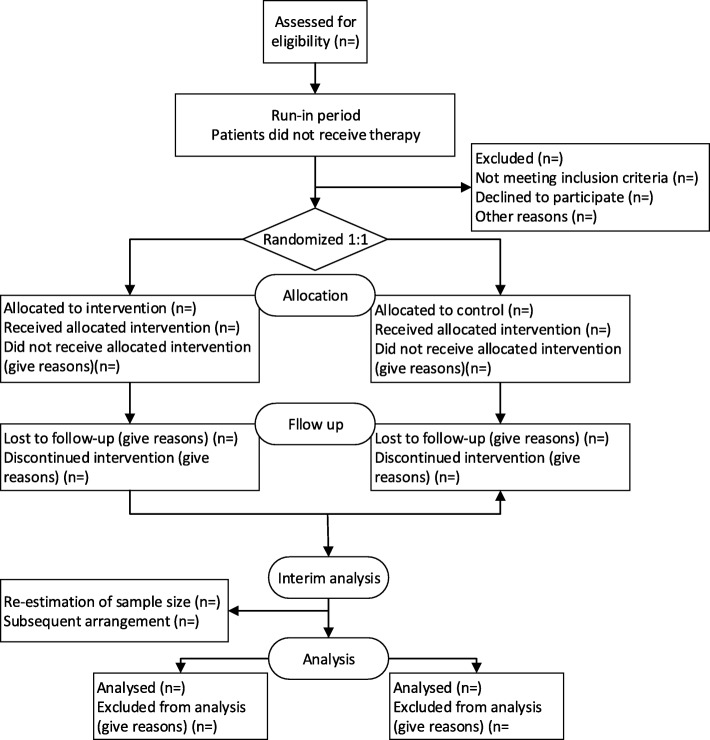
Trial flowchart

**Fig. 2:**
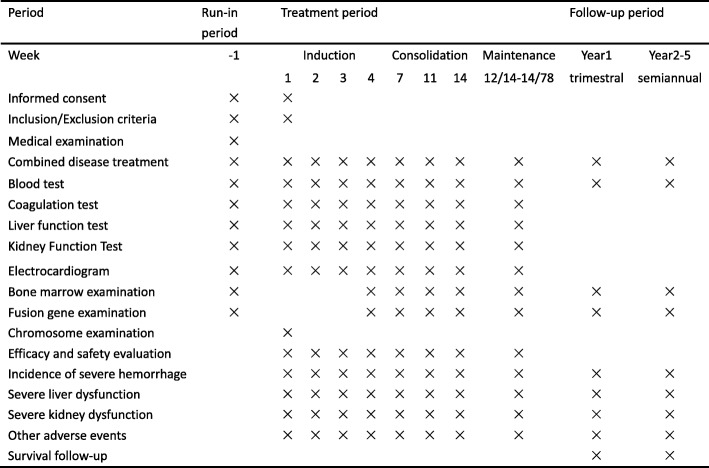
Trial process chart

All eligible patients who consent to participate will be randomized into either the experimental group or the control group in a 1:1 ratio. Randomization will be conducted using a clinical information management system (Figs. 1 and 2).

### Blinding

No blinding method is used in this experiment.

### Interventions

**Fig. 3:**
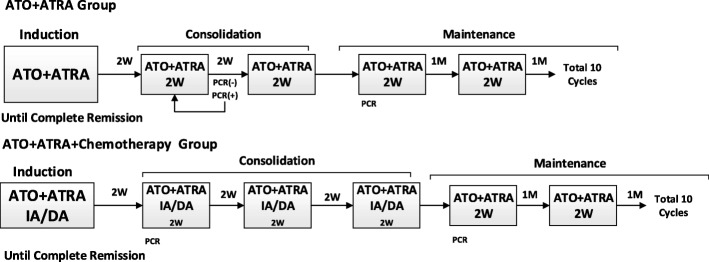
Treatment groups

Patients with APL were randomly assigned by using a computer-generated, random-allocation schedule into two groups: the ATRA-ATO group (arm A); the ATRA-ATO plus chemotherapy group (arm B). (Patients with APL will be classified into three risk categories on the basis of WBC count and platelet count. Low risk is a WBC count < 10 × 10^9^/L and a platelet count > 40 × 10^9^/L; intermediate risk is a WBC count < 10 × 10^9^/L and a platelet count < 40 × 10^9^/L; high risk is a WBC count > 10 × 10^9^/L.) (Fig. 3).

### Arm A (ATRA-ATO group)

#### Induction therapy

ATRA (20–45 mg/m^2^/day) plus ATO (0.15 mg/kg/day), which will be maintained until complete hematologic remission.

#### Consolidation therapy

ATRA plus ATO 2 weeks on and 2 weeks off, until *PML-RARα* is negative by reverse-transcriptase polymerase-chain-reaction (RT-PCR).

#### Maintenance therapy

ATRA plus ATO 2 weeks on and 1 month off, for a total of 10 courses. Detection of the *PML-RARα* fusion gene by RT-PCR assay every 3 months will also be carried out.

The high-risk patients (arm A) will receive mannitol treatment when using ATO [9]. From the third week of induction treatment on, the daily protocol was as follows: a bolus infusion of 125 mL 20% mannitol administered intravenously via the antecubital vein in about 10 min total; a slow intravenous infusion of 125 mL 20% mannitol plus ATO in 500 mL physiological saline (NS) in about 4 h. Urine flow is measured to ensure maintenance of a rate of at least 30–50 mL/h.

Guidelines for administering hydroxyurea in patients (arm A) who developed leukocytosis after initiation of therapy are detailed in the table below (Table 1)

**Table 1:** Guideline for administering hydroxyurea

White blood cell (WBC) counts	Dose of hydroxyurea
5–10 × 10^9^/L	0–-1 g/day
10–20 × 10^9^/L	1.0–2.0 g/day
20–40 × 10^9^/L	2.0–4.0 g/day
≥ 40 × 10^9^/L	4.0 g/day

### Arm B (ATRA-ATO plus chemotherapy group)

#### Induction therapy

ATRA (20–45 mg/m^2^/day) plus ATO (0.15 mg/kg/day), until complete hematologic remission. At the same time, idarubicin (8 mg/m^2^/day), given on days 2, 4, 6; *or* DNR (25–45 mg/m^2^/day), given on days 2, 4, 6, 8 plus Arc-C (150 mg/m^2^/day), given on days 1–7.

#### Consolidation therapy

Patients proceed to receive three courses consisting of ATRA and ATO 2 weeks on and 2 weeks off and anthracyclines on days 1–3 and Ara-C on days 1–7.

#### Maintenance therapy

- Non-high-risk: ATRA (20–45 mg/m^2^/day) plus ATO (0.15 mg/kg/day) 2 weeks on and 1 month off, for a total of 10 cycles
- High-risk: ATRA (20–45 mg/m^2^/day) plus ATO (0.15 mg/kg/day) 2 weeks on and 1 month off, for a total of 10 cycles. Orally administered methotrexate (MTX) (15 mg/m^2^/day) weekly 4 weeks on and 4 weeks off, for a total of 10 cycles; *or* 6-mercaptopurine (6-MP) (50 mg/m^2^/day) 4 weeks on and 4 weeks off, for a total of 10 cycles

Regarding management of differentiation syndrome (DS) for the two groups: at the earliest manifestations of suspected differentiation syndrome, intravenously administered dexamethasone is administered at a dose of 10 mg every 12 h until the disappearance of signs and symptoms for a minimum of 3 days.

### Monitoring

#### Follow-up monitoring

PCR should be performed on a bone marrow sample at the consolidation and maintenance therapy stages. We should monitor bone marrow samples by PCR every 3 months in the first year after finishing the maintenance therapy to detect molecular relapse. In the second year, the monitoring period can extend to 6 months. The sustained observation should last for 5 years. If the *PML-RARα* gene expression is positive, repeat PCR for confirmation within 4 weeks. If the *PML-RARα* gene expression is negative, the patients need to proceed to maintenance therapy or, conversely, to treat the patient for relapse.

#### Data monitoring

The study progress, safety data and data quality will be monitored by The Clinical Research Center of the First Affiliated Hospital of Xi’an Jiaotong University. The Clinical Research Center is an independent group of experts who monitor patient safety and treatment efficacy data while the clinical trial is ongoing. After each interim analysis, the Data Monitoring Committee will determine whether it is necessary to continue, modify or terminate the collection of these outcome data.

### Harms

Every hyperleukocytosis and QT-interval prolongation occurring during the study must be recorded. The following information should be recorded: occurrence time, severity, duration, adopted measure, and the outcome of the adverse event.

### Baseline characteristics

Baseline characteristics in each group will be analyzed using descriptive statistics, including means or medians for continuous variables and percentages for categorical variables.

### Outcome measures

In this trial, we selected 2-year DFS rates as the primary outcome. The secondary outcomes of this trial include complete remission (CR) rate and treatment-related mortality.

### Safety and adverse events

Any serious adverse events that occur in the study period should be recorded. If adverse events occur, the investigator will determine whether the participant should withdraw from the study, according to the condition of the patient. For serious adverse events, the investigator must immediately take necessary measures and report to the investigator and the Ethics Committee. Serious adverse device effects that are still ongoing at the end of the study period must be followed up to determine the final outcome.

### Data collection and management

Patient information will be added into the paper case report form (CRF) promptly and synchronously with input into the electronic CRF. The occurrence of unexpected problems during this process should be recorded, and will be informed in a timely manner.

To ensure the effectiveness and integrity of the trial design, we use the committee who were responsible for the study design and the implementation process. They will supervise the integrity and accuracy of data collection to control its quality. The committee also needs to evaluate key outcomes based on their clinical expertise.

Data sharing is not applicable to this protocol article as no datasets have yet been generated or analyzed during the current study. The results should be made public within 24 months of reaching the end of the study. The end of the study is the time point at which the last data items are to be reported, or after the data are sufficiently mature for analysis as defined in the “Statistical analysis” section. A full report of the outcomes should be made public no later than 3 years after the end of the study. Results will also be available through Chinese Clinical Trials Registry.

### Statistical analysis

Statistical analysis will be performed using Statistical Analysis System version 18.0.

Descriptive analysis (calculations of averages, frequencies, proportions or rates) was conducted. The *t* test and Mann-Whitney *U* test will be used to detect differences in the distribution of continuous parametric and non-parametric variables, respectively. Chi-square or Fisher’s exact tests will be used for comparison of categorical outcomes. The survival functions will be estimated by using the Kaplan-Meier method and compared using the log-rank test. All statistical tests will be two-sided, and the level of significance will be set at 0.05 except for the non-inferiority hypothesis.

### SPIRIT

This protocol has been written in accordance with the Standard Protocol Items: Recommendations for Interventional Trials (SPIRIT) guidelines. The SPIRIT Checklist is in Additional file 1.

## Discussion

Iland et al [10, 11] showed that ATRA-ATO plus chemotherapy is superior to ATRA plus chemotherapy in all-risk APL patients. The APL0406 study indicates that ATRA-ATO is superior to ATRA chemotherapy in non-high-risk APL patients [6]. The necessary use of ATO has been proved by many studies. But there is no randomized trial to compare which is more efficacious and safe between ATRA-ATO and ATRA-ATO plus chemotherapy in all-risk patients. Hence, our trial is to compare an ATRA-ATO group versus an ATRA-ATO with chemotherapy group in all-risk patients. Through this trial, we hope to reach a conclusion as to whether chemotherapy is necessary for all-risk patients.

The optimal therapy for high-risk patients remains unclear. Burnett et al [8] found that ATO and ATRA is a feasible treatment not only in low-risk but also in high-risk patients. The high-risk patients received a dose of the immunoconjugate gemtuzumab ozogamicin to decrease the peripheral WBC count. However, immunoconjugate gemtuzumab ozogamicin is not available in many countries. Our study included the high-risk patients, who are known to have higher initial WBC counts and a higher rate of early mortality. Differentiation syndrome is the most serious complication of ATRA-ATO therapy and is related to leukocytosis during induction therapy [12, 13]. Hence, taking control of the peripheral WBC count may effectively reduce the mortality of APL patients and reduce the complications of APL patients. In the experimental group, we use hydroxyurea to reduce the peripheral WBC count and decrease the risk of the APL differentiation syndrome.

Patients with leukocytosis may have a high risk of CNS relapse [14]. The first prevention measure is to decrease the peripheral WBC count by hydroxyurea or chemotherapy. It is difficult to prevent CNS relapse because the therapeutic drugs do not easily penetrate the BBB. So, the second prevention measure could be to increase the permeability of ATO by mannitol [15]. Hyperleukocytosis remains the most frequent cause of treatment failure. Daver et al. [16] show that patients with hyperleukocytosis had inferior CR rates (69% versus 88%; *P* = 0.004) and higher 4-week mortality (24% versus 9%; *P* = 0.018) compared to patients without hyperleukocytosis. Through a retrospective study, we analyzed the relationship of mortality with the peripheral WBC count in 117 APL patients over 8 years. We found that the highest value of the peripheral WBC count was related to the mortality of patients with APL. For patient safety, we only recruit the patients whose peripheral blood WBC count is < 40 × 10^9^/L at diagnosis. This protocol is simple and convenient to implement.

### Trial status

Recruitment commenced in July 2015, and the trial will schedule to end in December 2018.

## Additional file

Additional file 1: Standard Protocol Items: Recommendations for Interventional Trials (SPIRIT) 2013 Checklist. (DOC 123 kb)

## Abbreviations

6-MP: 6-mercaptopurine
APL: Acute promyelocytic leukemia
ATO: Arsenic trioxide
ATRA: All-trans retinoic acid
BBB: Blood-brain barrier
CNS: Central nervous system
CR: Complete remission
CSF: Cerebrospinal fluid
DS: Differentiation syndrome
MTX: Methotrexate
NS: Normal saline
RT-PCR: Reverse-transcriptase polymerase-chain-reaction
